# Infectious disease consultations at a South African academic hospital: A 6-month assessment of inpatient consultations

**DOI:** 10.4102/sajid.v35i1.169

**Published:** 2020-09-09

**Authors:** Lauren Richards, David C. Spencer, Jeremy S. Nel, Prudence Ive

**Affiliations:** 1 Division of Infectious Diseases, Department of Medicine, Faculty of Health Sciences, Helen Joseph Hospital, University of the Witwatersrand, Johannesburg, South Africa; 2 Clinical HIV Research Unit (CHRU), University of the Witwatersrand, Johannesburg, South Africa

**Keywords:** infectious diseases, inpatient, consultations, South Africa, HIV, TB

## Abstract

**Background:**

Infectious diseases (IDs) dominate the disease profile in South Africa (SA) and the ID department is increasingly valuable. There has been little evaluation of the IDs consultation services in SA hospitals.

**Methods:**

A qualitative review of ID inpatient consultations was performed over 6 months at a SA tertiary hospital. Prospectively entered data from each consultation were recorded on a computerised database and retrospectively analysed.

**Results:**

749 ID consultations were analysed, 4.8% of hospital admissions. Most consultations included initiation of antiretroviral therapy (ART) (27.8%), lipoarabinomannan antigen testing (24.8%) and change of ART (21.6%). Of patients reviewed, 93.3% were human immunodeficiency virus (HIV) positive and the median CD4 count was 52 cells/mm^3^. The infectious diagnoses (excluding HIV) most frequently encountered were pulmonary and abdominal tuberculosis (TB) and acute gastroenteritis. When all subcategories of TB infection were combined, 42.9% were found to have TB. Patients had predominantly one (45.4%) or two (30.2%) infectious diagnoses in addition to HIV. Some (12%) had three infectious diagnoses during their admission. The number of diagnoses, both infectious (odds ratio [OR] 2.00; 95% confidence interval [CI] 1.11–3.60) and non-infectious (OR 2.27; 95% CI 1.25–4.11), was associated with increased odds of death.

**Conclusion:**

The IDs department sees a high volume of patients compared to most developed countries. HIV, TB and their management dominate the workload. This study shows that HIV patients still have significant morbidity and mortality. The complexity of these patients indicates that specific expertise is required beyond that of the general physician.

## Introduction

Infectious diseases (IDs) is a subspecialty of internal medicine that is relatively new in South African (SA) medicine because the specialty had its first graduates only in 2004.^1^ The value of a physician specially trained in IDs and the management thereof is becoming increasingly apparent as ID dominates the SA disease profile.^2^ Specialist ID consultation has been shown to decrease mortality, improve patient outcomes and reduce expenditure on care.^3,4,5,6,7^

Antimicrobial resistance threatens the ability to manage common IDs and this has been exacerbated by the paucity of new antimicrobial agents. These two factors underpin the urgent need for antibiotic stewardship. In this regard, ID specialist intervention improves the appropriateness of the antibiotic prescribed, the dose and the duration. As such, these specialists are in the vanguard of antibiotic stewardship programmes.^3,4,5,6,7^ Pulcini et al. reviewed 31 studies from around the world evaluating the effect of ID specialists on antimicrobial prescriptions in hospitals.^5^ The appropriateness of the antibiotics prescribed was improved in almost all of the studies reviewed across a range of diseases, clinical settings and types of antimicrobials used.

Aside from increased resistance to antibiotics, ID specialists are becoming more relevant as the principal therapists of an increasing population of immunocompromised patients. This is of particular importance in South Africa, where although according to Statistics South Africa, in 2015, 11.2% of the SA population was human immunodeficiency virus (HIV)-positive, the average life expectancy of SAs had risen to 62 years. This increase in life expectancy is in part attributed to a decrease in the number of deaths because of the acquired immunodeficiency syndrome (AIDS), primarily because of the increased use of antiretroviral therapy (ART).^8,9^ As the HIV-positive population lives longer, new medical challenges arise, increasing the need for specialists in this field.^10^

In addition to an ageing HIV population, there are still a large number of HIV-positive patients not yet on ART who are presenting to healthcare facilities at a late stage of their illness with infections, opportunistic or otherwise.^11^ This frequently represents significant diagnostic difficulty as they may have more than one disease process or may present atypically. Managing this type of patient is often complex, involving the initial diagnosis and therapy, the initiation of ART and monitoring for drug interactions and side effects. The management of patients with HIV requires specialist knowledge and training for which the ID specialist is eminently qualified.

In many SA hospitals, ID specialist input is mainly in the form of bedside consultations, as typically there is no dedicated ID ward. This makes evaluation of the role of ID specialists more difficult. To date, there is a paucity of information on the spectrum of patients and problems that SA ID departments encounter. This review may assist in highlighting the type of patients typically referred, which may also indicate the areas that need further attention or improvement and define where further education is necessary. As South Africa has a unique patient population, particularly in relation to the developed world, this review may cast further light on the current state of IDs in a developing country.

## Methods and design

This is a retrospective descriptive review of all formal ID consultations requested during a period of 6 months. The site of the review is the Helen Joseph Hospital (HJH) located in Johannesburg, South Africa. The Helen Joseph Hospital is a tertiary academic hospital affiliated with the University of the Witwatersrand. During the study period, the IDs department consisted of one full-time ID specialist physician, one ID fellow, two medical registrars and one medical officer. There is no specific ID ward and, therefore, the department works on a consultation basis.

From October 2015, all consecutive, inpatient IDs consultations were captured on a computerised database by the medical registrar or medical officer performing the consultation. All consultations performed by the IDs department over a 6-month period (October–March 2016) were analysed and made up the study population. Consultation details recorded included patient’s gender, age, time period between hospital admission and ID consultation, the reason for the ID consultation, HIV status, ‘CD4 T lymphocyte’ (CD4) count if the patient was HIV-positive, the main infectious diagnoses, the non-infectious comorbidities and patient outcome. Outcome was defined as death (if the patient demised during the period of consultation) or discharge (if the patient was still alive at the time of discharge from ID services). The CD4 count was recorded as an absolute number and sorted into three categories (≤ 100, 101–500 and > 500). The cut-off value of 100 cells/mm^3^ was chosen as this implies that a patient is at risk of specific opportunistic infections that rarely occur otherwise. The cut-off of 500 cells/mm^3^ was chosen as this was the number at which South Africa initiated ART during the review period; this has since changed. Only formal bedside consultations, requested via a referral letter, were included and telephonic or informal consults were excluded. Repeat visits by the ID team during a patient’s admission were not recorded as additional consultations. Sample size estimation was based on the key research question to be answered, in this case the estimation of proportions (e.g. the proportion of women in the study group). Based on worst-case (for sample size) estimates of 50%, precision of 5% and the confidence level of 95%, a sample size of 385 would be required. The actual sample size of 749 in this study is thus more than adequate and corresponds to a precision of 3.6% (rather than 5%).

Data analysis included the previously mentioned consultation details. Categorical variables were summarised by frequency and percentage tabulation. Continuous variables were summarised by the mean, standard deviation, median and interquartile range (IQR). In addition, the number of deaths per 100 consultations and the number of total hospital admissions requiring an ID consultation per month were calculated. Lastly, an attempt to associate a patient’s length of stay (≤ 10 days vs. > 10 days) with outcome was assessed by logistic regression and odds ratio (OR), controlling for HIV status or CD4 count category, number of infectious diagnoses (0–1 vs. more than 1) and number of non-infectious diagnoses (0 vs. 1 or more). Analysis was performed using statistical analysis system (SAS) (version 9.4 for Windows). A *p*-value of < 0.05 indicates significant associations.

### Ethical consideration

Ethical clearance was obtained from the University of the Witwatersrand Human Research Ethics committee (clearance certificate number is M160486).

## Results

During the 6-month study period, a total of 15 472 patients were admitted to HJH (October 2015–March 2016) (Table 1). Of these, 749 (4.8%) were seen by ID as inpatient consultations. There was an increase in the proportion of consultations over the course of the study, from 3.6% at the start to 5.2% at the end. The median percentage of all hospital admissions seen by the IDs department as formal inpatient consultations was 4.9% (IQR 4.38–5.35). These inpatient consultations were analysed: the study group consisted of 50.9% men and 49.1% women. The male to female ratio was 1:04. The median age of the patients was 38.8 years (IQR 32.8–47 years). The median length of stay of a patient prior to an ID consultation was 6 days (IQR 3–9 days; range 0–114 days) and 19.6% of the patients received the ID consultation after more than 10 days. There was no significant association between length of stay prior to consultation and patient outcome, given the other variables in the model (OR 1.24; 95% CI 0.63–2.42) (Table 2).

**TABLE 1:** Monthly infectious disease consultations as a percentage of total hospital admissions at Helen Joseph Hospital.

Month and year	Total admissions	ID consultations	ID consultations (%)
October 2015	3232	117	3.6
November 2015	2998	130	4.3
December 2015	2570	119	4.6
January 2016	2199	119	5.4
February 2016	2309	152	6.6
March 2016	2164	112	5.2

**Total**	**15 472**	**749**	**4.8**

ID, infectious disease.

**TABLE 2:** Variables and their impact on patient outcome amongst patients seen by the infectious diseases department at Helen Joseph Hospital from October 2015 to March 2016.

Variable	Effect	*p*	Odds ratio	95% confidence limits for odds ratio	
				*n*	%
LOS prior to consultation	> 10 d vs. 0–10 d	0.53	1.24	0.63	2.42
HIV status or CD4 count	CD4 ≤ 100 vs. HIV-negative	0.29	2.21	0.51	9.70
	CD4 101–500 vs. HIV-negative	0.78	1.24	0.26	5.86
	CD4 > 500 vs. HIV-negative	0.87	0.82	0.07	9.55
Number of infectious diagnoses	More than 1 vs. 0–1	0.020	2.00	1.11	3.60
Number of non-infectious diagnoses	1 or more vs. 0	0.0069	2.27	1.25	4.11

d, days; HIV, human immunodeficiency virus; LOS, length of stay; vs., versus.

**FIGURE 1:**
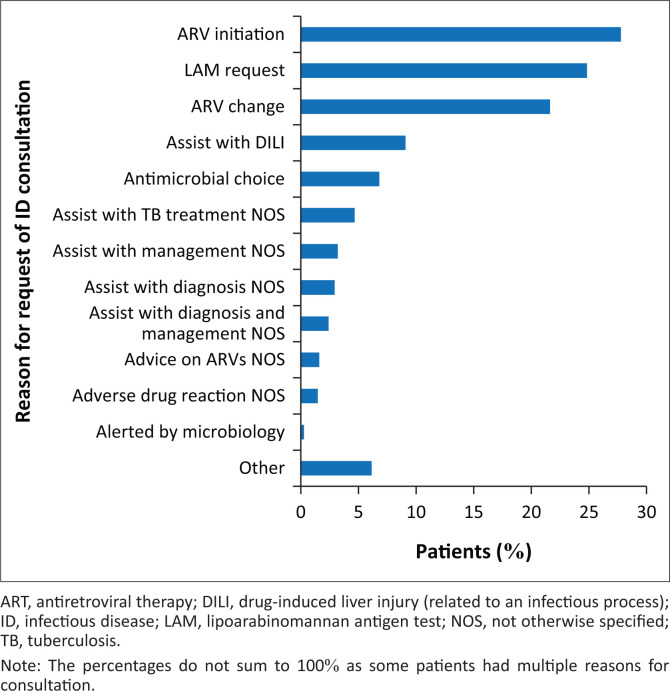
Reason for request of an infectious disease consultation amongst inpatients at Helen Joseph Hospital during October 2015 – March 2016 (*N* = 749).

The most common reasons for consultation were ART initiation (27.8%), lipoarabinomannan antigen (LAM) testing (24.8%), ART change (21.6%), assistance in managing a drug-induced liver injury (DILI) (9.1%) and assistance in antimicrobial choice (6.8%). Some consultations were not requested by treating doctors but were requested by microbiology as they felt an ID consultation would benefit the patient (Figure 1).

Of those patients with a known HIV status (*n* = 741), 93.3% (*n* = 691) were HIV-positive. The median CD4 count of these 691 patients was 52 cells/mm^3^ (IQR 15–54; range 0–1125) (*n* = 682; 1.3% missing data). When CD4 counts were categorised, the majority of patients (65.5%) had CD4 counts of 100 cells/mm^3^ or less.

**FIGURE 2:**
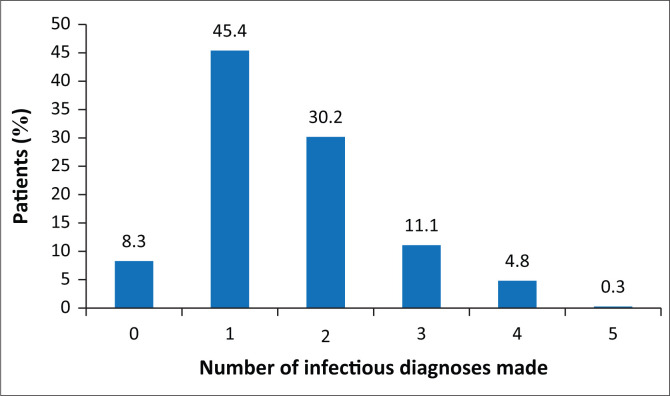
Number of infectious diagnoses (excluding human immunodeficiency virus) made in individual patients seen by infectious diseases department at Helen Joseph Hospital (October 2015 – March 2016).

In terms of diagnoses, 91.7% of the patients had at least one infectious diagnosis (excluding HIV), whilst 51.9% had at least one non-infectious diagnosis. Patients had predominantly one (45.4%) or two (30.2%) infectious diagnoses in addition to HIV. A certain proportion of patients (12%) had three infectious diagnoses made during the admission (Figure 2).

**FIGURE 3:**
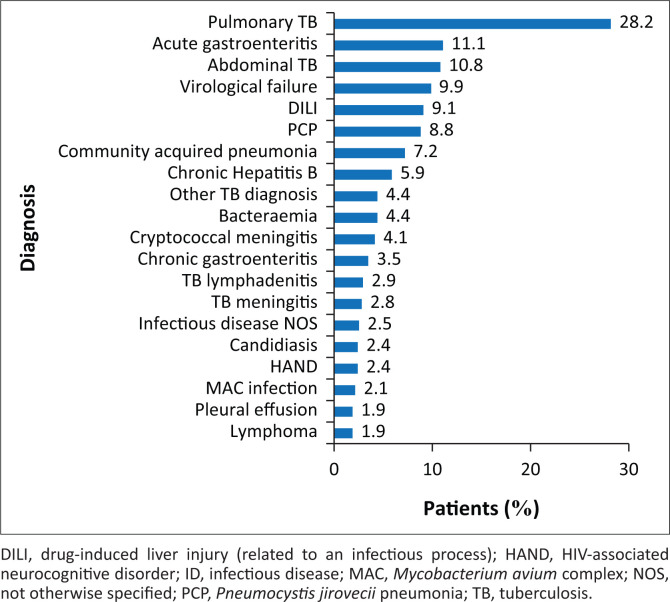
Top 20 infectious diagnoses (excluding human immunodeficiency virus) made in individual patients seen by infectious diseases department at Helen Joseph Hospital during October 2015 – March 2016.

**FIGURE 4:**
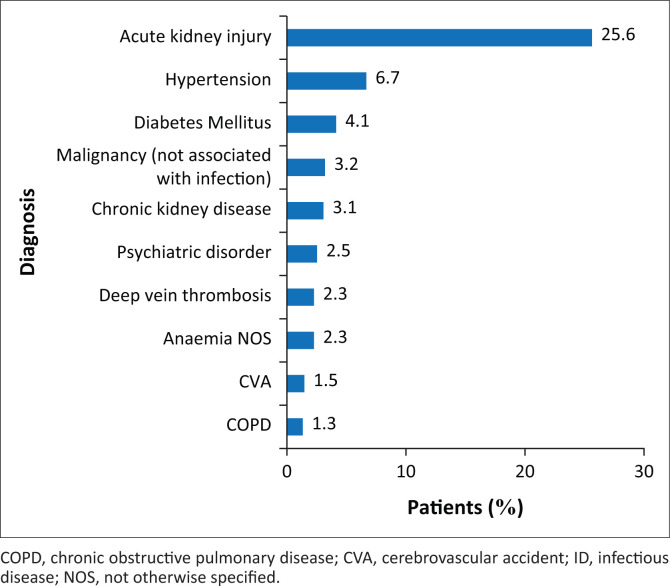
Top 10 non-infectious co-morbidities made in individual patients seen by infectious diseases department at Helen Joseph Hospital during October 2015 – March 2016.

The most common infectious diagnosis was pulmonary TB followed by acute gastroenteritis, abdominal TB, virological failure on ART and DILI (Figure 3). When combining all subcategories of TB infection, a total of 42.9% of patients were infected with TB. Acute kidney injury was the most common non-infectious diagnosis followed by hypertension, diabetes mellitus (DM), non-AIDS-defining malignancy and chronic kidney disease (Figure 4). Of the patients seen, 59 (7.9%) died, whilst the ID team was still in attendance. More than one infectious diagnosis increased the odds of death compared to no or one infectious diagnosis, given the other variables in the model (OR 2.00; 95% CI 1.11–3.60) (Table 2). One or more non-infectious diagnoses increased the odds of death compared to no non-infectious diagnosis, given the other variables in the model (OR 2.27; 95% CI 1.25–4.11) (Table 2).

## Discussion

South Africa is still the epicentre of the HIV epidemic. This creates a disease and patient profile that is very different from that found in developed countries.^12^ This review has described the burden of disease that the ID specialist faces in a tertiary hospital in South Africa. It has demonstrated the complexity of the patients consulted and the impact of an ID physician on patient care. To my knowledge, this review, together with another study performed by Pandie et al.^2^ at Groote Schuur Hospital in Cape Town, provides the only available data on the functioning of SA ID units.

The HJH ID unit sees 4.8% of total hospital admissions, which is significantly greater than that of hospitals outside of South Africa.^13,14,15^ This may be attributed to the hospital policy that all patients initiating ART must have an ID consultation; however, even after these patients were excluded, 3.7% of all admissions still required a consultation. Other reasons to consider are the possibility that there is a higher proportion of infectious diagnoses amongst total admissions to HJH or that in general patients who are admitted may be sicker. As most of the patients seen by the ID unit were HIV-positive with relatively low CD4 counts, this is a plausible explanation. Another reason for the number of consults could be the perception that the ID unit could be of assistance in accelerating or facilitating access to certain investigations, which may aid diagnosis, and expedite discharge.

In this study, there was an increase in the proportion of consultations requested from the start to end of the review that was not accounted for by changes in hospital admissions. One explanation for this could be improved data collection over time, as the implementation of the database and the start of the review coincided. As medical registrars and medical officers responsible for data capturing became more familiar with the process, they may have seen the benefits and become more involved. Alternatively, the IDs department may have gradually been receiving more referrals over the study period.

Of the total number of patients seen, 93.3% were HIV-positive. Two-thirds of these had CD4 counts of less than 100 cells/mm^3^. The median CD4 count of all HIV-positive patients was 52 cells/mm^3^. This is striking as it shows that patients are still presenting to healthcare facilities at an advanced stage of their illness despite increased availability of ART initiation centres in South Africa. HJH ID unit is no exception in this regard: the review by Pandie et al.^2^ found that 80% of patients seen by the ID unit at Groote Schuur Hospital were HIV-positive, with a median CD4 count of 128 cells/mm^3^. An editorial commentary by Ford et al. stated that the current European consensus definition of late presenters is a patient with ‘a CD4 cell count of < 350 cells/mm^3^ or an AIDS diagnosis within 6 months of HIV diagnosis’.^11^ As such, the vast majority of patients initiating ART in sub-Saharan Africa are late presenters and caring for these sick patients constituted a significant portion of the workload of the ID specialist.^11^

The most common reason for ID consultation in this review was for ART initiation (27.8%). As stated above, this is, in part, because of the policy at HJH requiring that all ART must be initiated by a member of the IDs department. However, another factor contributing to this is that most of these patients were eligible for ART initiation as at the time of the review ARTs were only initiated at CD4 counts ≤ 500 cells/mm^3^. Assistance with changing ART, for a variety of reasons, closely followed ART initiation as the third most common reason for consultation. These data clearly reflect the HIV burden experienced in South Africa and emphasise the complexities of management of these patients, and as such often needing the assistance of a specialist.^16^

The complexity of these patients, contrary to the principle espoused in Occam’s razor (that ‘plurality must not be posited without necessity’), was exaggerated by the fact that most patients were found to have multiple diagnoses, both infectious and non-infectious in nature.^17^ Excluding the HIV positivity, 45.4% of the patients in this review had one, 30.2% had two and 12% had three distinct and active infectious diagnoses. Just less than half of the patients had at least one infectious diagnosis (excluding HIV) together with one non-infectious co-morbidity. Human immunodeficiency virus-infected individuals are at an increased risk of acquiring diseases historically associated with advanced age and half of the patients in this review had at least one non-infectious co-morbidity. Acute kidney injury was diagnosed in 25% of patients and chronic kidney disease was the fifth most common co-morbidity. The data suggest that there is a heightened risk of kidney disease amongst SAs with advanced HIV infection in keeping with current literature. Thereafter the most prevalent non-infectious diagnoses were hypertension, DM and all forms of malignancy.^18^ There is conflicting evidence as to whether HIV is an independent risk factor for developing DM, but certain complications associated with DM can be seen more commonly in HIV-positive patients.^19,20,21^ Human immunodeficiency virus is changing the assumption that a single diagnosis, infectious or otherwise, can alone explain the clinical picture, especially at very low CD4 counts. Failure of the treating physician to recognise this is likely to result in inadequate medical management and the failure of the patient to respond to medical care.

The HIV epidemic has brought with it an explosion of TB. Tuberculosis was the most common diagnosis amongst HIV-positive and seronegative patients in the review by Pandie et al.^2^ The review by Meintjes et al. reported that the most common admission diagnosis amongst HIV-positive individuals at an SA district hospital was TB, and it was also the most common cause of death.^22^ Similarly, the most frequent infectious diagnosis made in this review aside from HIV was TB. When all forms of TB were taken as a collective, 42.9% of patients seen by the ID unit had this diagnosis. Interestingly, the second most frequent reason for a consult was to gain access to the test for the mycobacterial LAM antigen, a urine-based investigation that assists in the diagnosis of disseminated TB, the use of which is controlled by the IDs department at HJH. The test is restricted as it is not freely available in the government sector and, therefore, it is in limited supply. It is further restricted to HIV-infected individuals with low CD4 counts in whom it is most sensitive and in whom the diagnosis cannot be made in any other way.^23^ The fact that it is the second most common reason for consultation illustrates how frequently the diagnosis of TB is suspected in our patient population and how difficult it can be to make a definitive diagnosis in the presence of immune compromise.^24^

Apart from HIV and TB, the ID department at HJH assists with antibiotic choice, which is a more traditional area of ID expertise particularly in the developed world.^14,25,26,27^ Assistance with antimicrobial choice was the fifth most common reason for requesting a consultation, and the infectious diagnoses most frequently encountered were acute gastroenteritis, community-acquired pneumonia and bacteraemia of unknown aetiology.

There were 7.9 deaths per 100 consultations during the review period. More than one infectious diagnosis (excluding the diagnosis of HIV) increased the odds of death compared to no or one infectious diagnosis. In terms of non-infectious diagnoses, one or more diagnoses also increased the odds of death relative to those without.

Limitations of this review include the fact that a number of different people were responsible for entering the data. There was no way to assess if all consultations were entered into the database and as such there may have been some for which data were not recorded. This review did not include informal or telephonic consultations, which also contribute to the workload of the IDs department. Another aspect that is not generally considered and adding to the workload are the follow-up visits of patients known to the department, which are not documented. Patients were followed up until hospital discharge, demise, recovery or to a point where it was felt that the department could no longer offer any assistance. The latter may have been because of a particularly poor prognosis where care was considered to be futile, which may have led to an underestimation of the overall mortality. Finally, the data available did not allow for an analysis of the cost-effectiveness of an ID consultation.

## Conclusion

In summary, sick HIV patients are still coming to medical attention with significant morbidity and mortality. Although TB is always the clinician’s number one concern, it should not be the only one. Practitioners need to consider other diagnoses, especially in patients who do not get better on TB treatment. Although these sick patients require ART, this is only part of the story. Achieving the Joint United Nations Programme on HIV/AIDS (UNAIDS) goal of the eradication of HIV by 2030 cannot be met only by supplying ART. The epidemic in Africa demands improvements in all facets of HIV management if this goal is ever to be met. The fact that the ID unit is so frequently requested to assist in these areas reflects the overall complexity of these cases such that specific expertise is required beyond that of the general practitioner or even the specialist physician. The ID physicians are best qualified to manage these patients and to educate their colleagues in the intricacies of care particularly with advanced HIV. Although this specialty is a relative newcomer, this study and others have proven the worth of the discipline.
